# Dataset reporting detection of breast cancer-related *HER2*^*I655V*^ polymorphism using allele-specific polymerase chain reaction

**DOI:** 10.1016/j.dib.2016.09.033

**Published:** 2016-09-24

**Authors:** Bugi Ratno Budiarto, Desriani

**Affiliations:** Research Center for Biotechnology, Indonesian Institute of Sciences (LIPI), Jalan Raya Bogor Km. 46, Cibinong 16911, Indonesia

**Keywords:** Allele-specific PCR, HER2, Ile655Val, Single nucleotide polymorphism, Breast cancer

## Abstract

The dataset presented in this article is related to the research article entitled “Detection of *HER2* Gene Polymorphism in Breast Cancer: PCR Optimization Study” (B.R. Budiarto, Desriani, 2016) [1] with some modification in primers used and in PCR optimization strategy to eliminate false-positive result that may occur in *HER2*^*I655V*^ polymorphism detection. Combining a new set of primers with PCR gradient, The allele-specific PCR well performs to detect all type of breast cancer-originated *HER2*^*I655V*^ genotypes. The validation of this method was done using Sanger DNA sequencing, offering an alternative tool for *HER2*^*I655V*^ polymorphism detection in another type of cancer.

**Specifications Table**

**Table:** 

Subject area	*Biology*
More specific subject area	*Molecular Biology, Cancer research*
Type of data	*Table and figure*
How data was acquired	*PCR, DNA sequencing and statistics analysis*
Data format	*Raw, analyzed*
Experimental factors	*DNA was extracted form frozen section of breast cancer tissue*
Experimental features	*Allele-Specific PCR using two spesific primer (Forward direction) and one common primer (revere direction) to detect HER2*^*I655V*^*polymorphism*
Data source location	*Research Center for Biotechnology, Indonesian Institute of Science (LIPI), Indonesia*
Data accessibility	*Data is available with this article*

**Value of the data**

• The data provide a straightforward strategy for clarifying the possibility of a false-positive amplification generated by allele-specific PCR.
• The data provide the technique to determine the proper dosage of DNA template used in allele-specific PCR for the success of *HER2*^*I655V*^genotyping.
• Data comparison of *HER2*^*I655V*^ polymorphism between our improved allele-specific PCR with Sanger DNA sequencing is provided in a way to validate the method easily.
• Allele frequency data for breast cancer –related *HER2*^*I655V*^ polymorphism can be used as a reference for researchers who conduct similar experiments for *HER2*^*I655V*^ polymorphism studies with breast cancer risk related to specific demography or other races.

## 1 Data

**Fig. 1:**
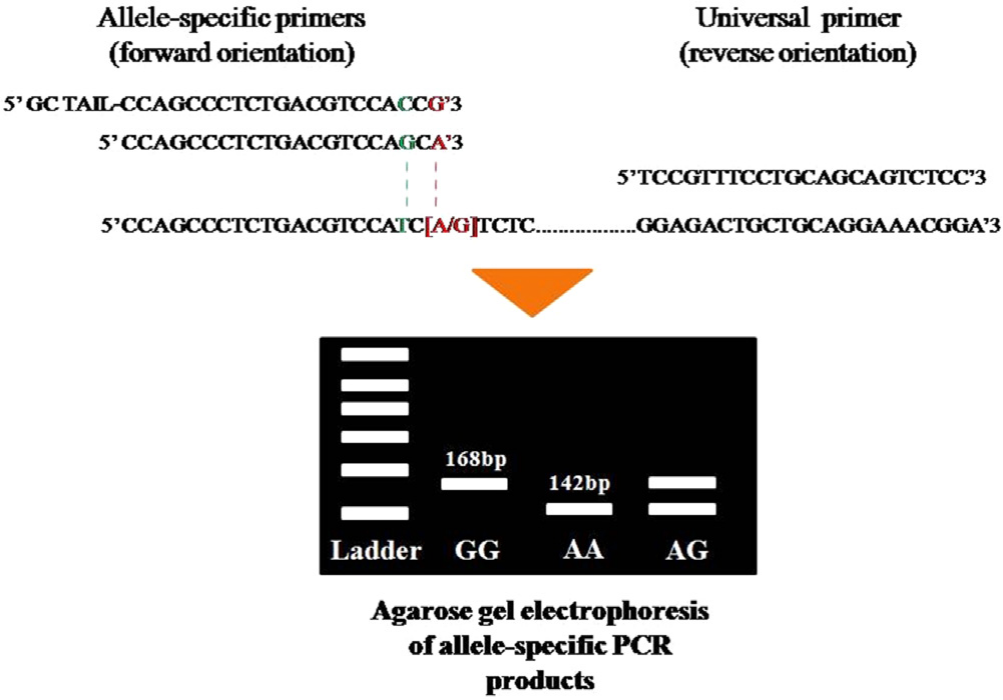
Illustration of allele-specific PCR and its primers design and position towards target *HER2*^*I655V*^ polymorphism. Nucleotides with red color at 3′ end of primer indicate the mismatched base pairing that correspond to SNP site. Nucleotides with green color at 3rd position indicate the mismatched base pairing to increase SNP discrimination level. (For interpretation of the references to color in this figure legend, the reader is referred to the web version of this article).

**Fig. 2:**
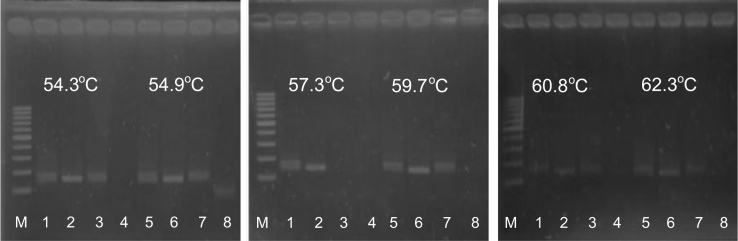
Determination of the best annealing temperature for allele-specific PCR in typing *HER2*^*I655V*^ polymorphism. Lane 1, 3, 5 and 7 are PCR products of AG genotype (142 bp and 168 bp), lane 2 and 6 are PCR products of AA genotype (142 bp), and lane 4 and 8 are NTC (Non-Template Control). No.6 genomic DNA was used as DNA template for AG genotype, while DEV genomic DNA was used as DNA template for AA genotype. M is 100 bp DNA ladder. The PCR product was run on 3% agarose under UV illumination.

**Fig. 3:**
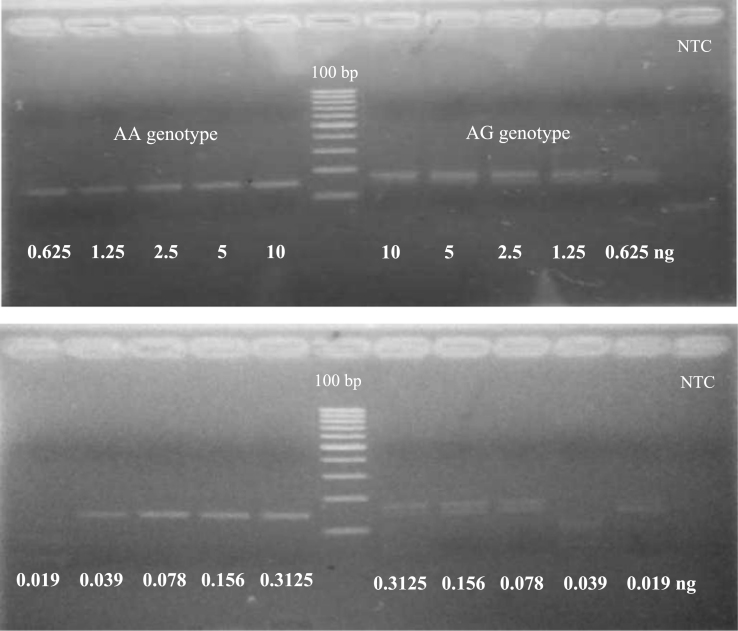
Sensitivity test of allele-specific PCR in genotyping *HER2*^*I655V*^ polymorphism. PCR was performed using optimized PCR condition where annealing temperature was at 54.3 °C. NTC was Non-Template Control. The PCR product was run on 3% agarose under UV illumination.

**Fig. 4:**
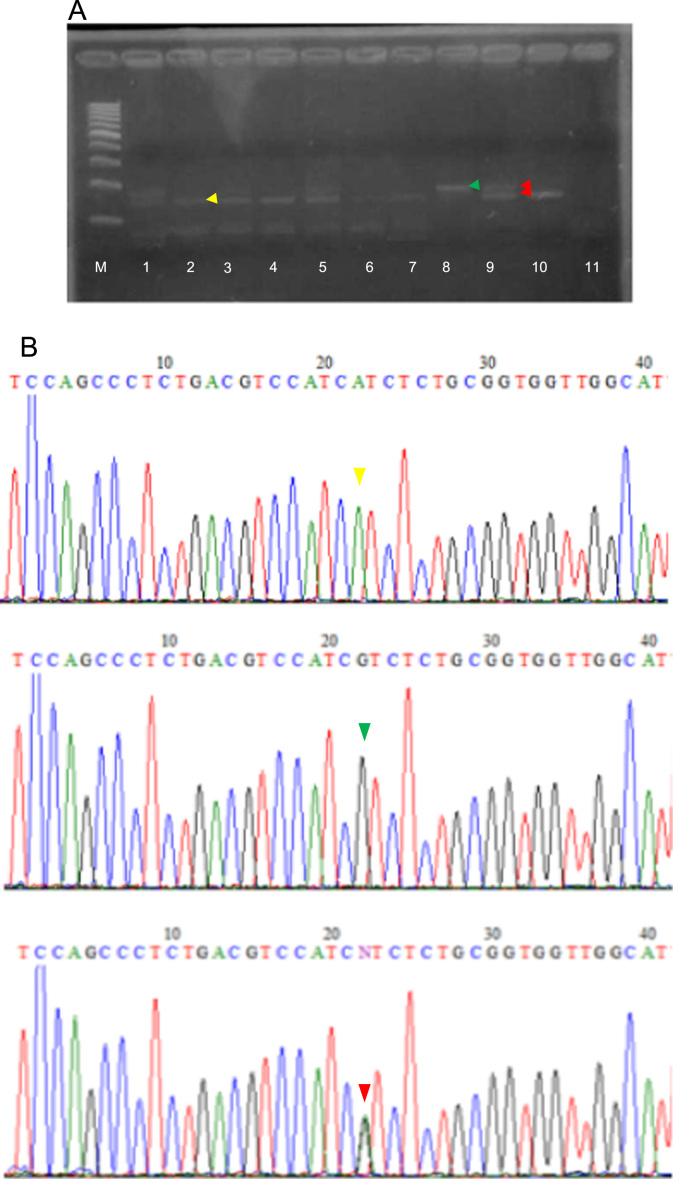
Allele-specific PCR of *HER2*^*I655V*^ polymorphism for 10 of 61 samples of breast cancer patients. (A) Gel electrophoresis data of allele-specific PCR: Yellow arrow head indicated AA genotype with band size of 142 bp; green arrow head indicated GG genotype with band size of 168 bp; red arrows head indicated AA/GG genotype represented two different of PCR band products. The PCR product was run on 3% agarose under UV illumination. M is 100 bp DNA ladder meanwhile NTC (Non-template control) was pointed in lane 11. (B) Sanger DNA sequencing chromatograms of *HER2*^*I655V*^ polymorphism. Yellow arrow head indicated AA genotype; green arrow head indicated GG genotype; red arrows head indicated AA/GG genotype. (For interpretation of the references to color in this figure legend, the reader is referred to the web version of this article).

The detection of breast cancer-related *HER2*^*I655V*^ polymorphism using allele-specific PCR using mismatch-specific primers strategy as shown in Fig. 1. The optimum annealing temperature for breast cancer-related *HER2*^*I655V*^ genotyping in allele-specific PCR was done using gradient PCR strategy with SNP-known DNA template (Fig. 2). To test sensitivity of the method, we used two types of genomic DNA which contain AA genotype or AG genotype in *HER2* gene as allele representative with diluted ranging from 0.019 up to 10 ng of the template at optimized PCR condition (Fig. 3). Then, the reliability of allele-specific PCR was compared with data of Sanger DNA sequencing by looking the consistency of *HER2*^*I655V*^ genotypes data between two methods (Fig. 4 and Table 1). The allele frequency of breast cancer-originated *HER2*^*I655V*^ obtained from allele-specific PCR was similar with an allele frequency data of breast cancer-originated *HER2*^*I655V*^ from Asian population (Table 2).

## 2 Experimental design, materials and methods

### 2.1 Sample collection and genomic DNA extraction

Sixty-one breast cancer tissues from archived biopsies as the frozen section were collected from M. Djamil Hospital Padang, West Sumatera Province. Genomic DNA was then extracted followed manual tissue DNA extraction protocol (Pure Link Genomic DNA Mini Kit; Invitrogen). We have also prepared two types of genomic DNA with SNPs have previously been determined using a Sanger DNA sequencing method. No.6 tissue-retrieved genomic DNA with *HER2* gene carries AG genotype while DEV (abbreviaton of patient׳s name) tissue-retrieved genomic DNA with *HER2* gene carries AA genotype. All subjects enrolled in this experiment were approved by the local ethics committee, issued by the Ministry of Health, Republic of Indonesia.

### 2.2 Allele-specific PCR optimization

The optimum annealing temperature for allele-specific PCR was determined using genomic DNA with known SNPs. Each of 12.5 µL reaction mixture was contained 11.25 µL of PCR Super Mix (Invitrogen), 0.1 µM of allele-specific forward primer (5′-CCAGCCCTCTGACGTCCAGCA-3′), 0.06 µM of long allele-specific forward primer (5′-GCGGGCAGGGCGGCGGGGGCGGGGCCCCAGCCCTCTGACGTCCACCG-3′), 0.15 µM of reverse primer (5′-TCCGTTTCCTGCAGCAGTCTCC-3′), and 0.25 µl (~3–6 ng) of DNA template. The Primers ratio used in this experiment followed the optimized PCR method as suggested by Germer and Higuchi, [2] and Gaundet et al. [3] with minor modification. The PCR amplification profile was as follows: initial denaturation at 95 °C for 5 min, followed by 35 cycles of denaturation at 95 °C for 20 s, gradient temperature annealing from 54.3 °C, 54.9 °C, 57.3 °C, 59.7 °C, 60.8 °C to 62.3 °C for 20 s, and extension at 72 °C for 30 s (Kyratec Super Cycler Thermal Cycler, Australia). All PCR tubes, distilled water, pipette tips, and pipettes were pre-treated by exposing them on UV-light for ±15-20 minute prior to use. All PCR reagent mixing was done under laminar air flow.

### 2.3 Sensitivity test of allele-specific PCR

Two genomic DNA with known SNPs (No.6 genomic DNA for AG genotype and DEV genomic DNA for AA genotype) were fixed at 50 ng/µL to make serial dilution ranging from 10 ng declining to 0.019 ng. These diluted DNA then were applied as a template for allele-specific PCR reaction at optimum annealing temperature with PCR reagents and PCR amplification profile followed method mentioned above.

### 2.4 Validating and genotyping test using allele-specific PCR

Sixty-one samples of breast cancer were tested for *HER2*^*I655V*^ polymorphism using optimized allele-specific PCR. The PCR condition and temperature profile were the same as mentioned above except for annealing temperature was fixed at 54.3 °C. The method was validated by direct Sanger DNA sequencing of 61 sample of breast cancer patients by firstly amplified DNA fragment using primer pairs *HER2*_F (5′-CCAGCCCTCTGACGTCCAT-3′) and *HER2*_R (5′-TCCGTTTCCTGCAGCAGTCTCC-3′) generating 142 bp PCR product. The PCR amplification profile was as follows: initial denaturation at 95 °C for 5 min, followed by 35 cycles of denaturation at 95 °C for 30 s, an nealing temperature at 60 °C for 30 s, and extension at 72 °C for 30 s. This PCR product was sequenced using *HER2*_R primer only done by First-Base Asia Ltd.

### 2.5 Comparing allele frequency of *HER*^*2I655V*^ obtained from our data to published data

Data were tested for statistical significance by using Statistical Package for the Social Sciences software system SPSS-17 statistical software (SPSS, Chicago,IL). Frequency of each *HER2*^*I655V*^ allele was calculated using formulation frequency of Ile=*f*(Ile/Ile)+0.5*f*(IleVal), whereas frequency of Val=*f*(Val/Val)+0.5*f* (IleVal). Chi-square test was used to analyze differences in genotype frequencies between our data with published dataset to evaluate any possible deviation from Hardy–Weinberg equilibrium of our genotyping data. Genotype frequencies are interpreted as statistically significant different if only the *P* value is less than 0.05 [4].

## Figures and Tables

**Table 1:** Distribution data of *HER2*^*I655V*^ polymorphism in two methods comparison.

**Allelic types**	**Methods**	
	**Sanger sequencing**	**Allele-specific PCR**
AA	37 (60.6%)	37 (60.6%)
GG	2 (3.3%)	2 (3.3%)
AG	22 (36.1%)	22(36.1%)
Failed	0 (0%)	0 (0%)
Total samples	61 (100%)	61 (100%)

Percentage means the number of samples produces correct genotype in total samples tested

**Table 2:** Genotype frequency of *HER2*^*I655V*^ polymorphism showed by our data versus published data.

Author	Year of publication	Genotypes (%)			Sample population	*P* value
		AA	GG	AG		
		Ile	Val			
Our experiment		60.6	3.3	36.1	Asian	
Allele frequency		**0.79**	**0.21**			
Xie et al. [5]	2000	71.7	3.2	25.1	Asian	>0.05^a^
An et al. [6]	2005	78.5	2.8	18.6	Asian	
Naidu et al. [7]	2008	71.7	3.4	24.7	Asian	
Allele frequency		**0.85**	**0.15**			
Keshava et al. [8]	2001	64.9	4.3	30.8	European	>0.05^b^
Millikan et al. [9]	2003	59.6	6.3	34.02	European	
Nelson et al. [10]	2005	58.2	5.6	36.2	European	
Allele frequency		**0.78**	**0.22**			
Millikan et al. [9]	2003	87.5	1.1	11.7	African	<0.05^c^
Siddig et al. [11]	2008	82.4	1.5	16.2	African	
kallel et al. [12]	2009	87.8	2.7	9.5	African	
Allele frequency		**0.92**	**0.08**			

**Note**: Each of *P* value represented the different of genotype frequency of *HER2*^*I655V*^ obtained from Chi-Square test.

a no siginificant event was observed between genotype frequency of *HER2*^*I655V*^ in our experiment with Asian population.

b no siginificant event was observed between genotype frequency of *HER2*^*I655V*^ in our experiment with European population.

c a siginificant event was observed between genotype frequency of *HER2*^*I655V*^ in our experiment with African population.
