# Supplementary material for: Dataset reporting detection of breast cancer-related HER2I655V polymorphism using allele-specific polymerase chain reaction
Source: Data Brief. 2016 Sep 24;9:689–95. doi: 10.1016/j.dib.2016.09.033 (PMC5072141; doi:10.1016/j.dib.2016.09.033)
Supplement: Supplementary file 1 — Supplementary material [file mmc1.docx]

The authors declare that we have no conflict of interest.

Cibinong, May 2016

Signature

Desriani


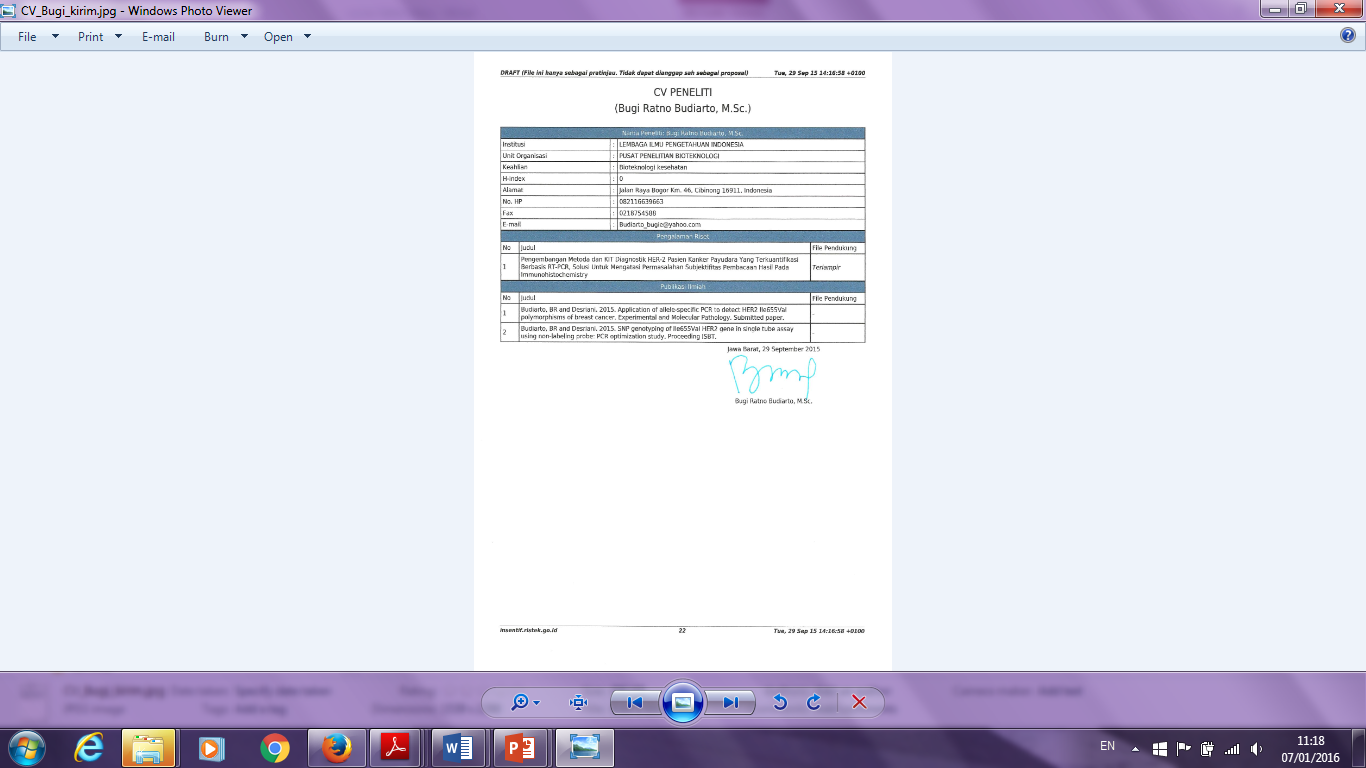


Bugi Ratno Budiarto
